# Profiles of Inflammatory Cytokines in the Vitreous Fluid from Patients with Rhegmatogenous Retinal Detachment and Their Correlations with Clinical Features

**DOI:** 10.1155/2016/4256183

**Published:** 2016-12-15

**Authors:** Shizuka Takahashi, Kobu Adachi, Yukihiko Suzuki, Atsuko Maeno, Mitsuru Nakazawa

**Affiliations:** Department of Ophthalmology, Hirosaki University Graduate School of Medicine, Hirosaki, Japan

## Abstract

*Purpose*. To characterize the profiles for inflammatory cytokines in the vitreous fluid from patients with rhegmatogenous retinal detachment (RRD) by comparing those of other vitreoretinal diseases and to analyze the correlation between intravitreal cytokines and clinical features.* Materials and Methods*. Vitreous fluid was obtained at the time of surgery from 28 RRD eyes. Vitreous fluid was similarly collected from patients with macular hole (MH), epiretinal membrane, proliferative diabetic retinopathy (PDR), and retinal vein occlusion as controls. Twenty-seven cytokines were measured. Intravitreal cytokine profiles in RRD were characterized by comparing these with those in other vitreoretinal diseases. We also analyzed the correlations between vitreous cytokines and clinical features.* Results*. There were statistical differences in the MCP-1, MIP-1*β*, and IP-10 between the RRD and MH, while the IL-6 and IL-8 exhibited levels that were between those for the PDR and MH. MIP-1*β* was significantly correlated to both the extent and duration of the RRD, while IL-8 was significantly correlated to the extent of the RRD.* Conclusions*. MCP-1, MIP-1*β*, and IP-10 may modify the pathologic features of RRD. The levels of these cytokines are related in part to the clinical features and the level of photoreceptor damage.

## 1. Introduction

Rhegmatogenous retinal detachment (RRD) is caused by the separation of the photoreceptor outer segments from the retinal pigment epithelium (RPE). This leads to a severe disturbance of the visual function corresponding to the detached area. In addition, the separation of photoreceptors from the RPE impairs the oxygen supply to the photoreceptor cells, which eventually induces photoreceptor cell death. Recent experimental studies have reported finding increased levels of inflammatory cytokines such as monocyte chemotactic protein-1 (MCP-1) and tumor necrosis factor *α* (TNF*α*) increased in the detached retina, which indicates that inflammatory reactions may modify the pathogenesis of the photoreceptor damage and the proliferative vitreoretinopathy (PVR) in RRD [1, 2]. More recently, Kunikata et al. demonstrated that the levels of MCP-1, macrophage inflammatory protein-1*β* (MIP-1*β*), and interferon-inducible 10 kDa protein (IP-10) were significantly increased in the aqueous humor of patients with RRD [3]. These authors also reported that administration of intravitreal injection of triamcinolone acetonide inhibited the increase of these cytokines [3]. Thus, the findings of their study confirmed the association between the inflammation and the pathogenesis of photoreceptor damage in RRD.

Previously, we demonstrated that not only vascular endothelial growth factor (VEGF) but also other inflammatory cytokines such as interleukin-8 (IL-8), MCP-1, MIP-1*β*, and IP-10 were elevated in the vitreous fluid of patients with proliferative diabetic retinopathy (PDR) and retinal vein occlusion (RVO) [4]. Based on these findings, we speculated that there were various cytokine networks associated with these pathologic conditions. Although there may be some similarities between RRD, PDR, and RVO with regard to intravitreal inflammatory cytokines such as MCP-1, IP-10, and IL-8 [5, 6], the specific differences and/or similarities in the expression profiles of the various cytokines for these diseases have yet to be definitively clarified.

Cytokines mediate various interactions between the cells for both physiologic and pathologic processes. By characterizing the expression levels of the various cytokines in RRD, this could potentially help us to achieve a better understanding of the pathogenesis of the photoreceptor damage, as well as providing clues for designing more effective treatments for photoreceptor protection in RRD. Therefore, after obtaining vitreous fluid from patients with RRD, the present study analyzed the expression profiles of various cytokines and then statistically compared these with the profiles determined for PDR, RVO, epiretinal membrane (ERM), and macular hole (MH). We also examined the interactions between several of the main cytokines that exhibited elevated levels in the vitreous fluid of RRD patients. In addition, we analyzed the relationships between the clinical features and the levels of these cytokines.

## 2. Subjects and Methods

The present study was performed in accordance with the tenets of the Declaration of Helsinki and approved by the Institutional Review Board of Hirosaki University Graduate School of Medicine. After receiving an explanation of the nature and possible consequences of taking part in the study, each patient provided informed consent.

### 2.1. Patients

During vitrectomy procedures that we performed at Hirosaki University Hospital, we used a previously described method to collect undiluted vitreous fluid from patients with RRD (28 eyes), PDR (55 eyes), RVO (10 eyes), ERM (9 eyes), or MH (14 eyes) [4]. Briefly, vitreous fluid (approximately 0.3 mL) was obtained from each eye prior to starting the fluid irrigation during the initial stage of the vitrectomy procedure. Vitreous samples were immediately cooled on ice in a dark container for approximately 1 to 2 h and then frozen at −80°C until analysis.

Background clinical data for the preoperative conditions were obtained from each of the patient's medical records. For RRD, in addition to patient age (61.2 ± 9.3 years old) and gender (male : female, 13 : 15), we also collected information on the extent of detachment (quadrants), duration of subjective symptom (weeks), vitreous status including the presence of vitreous hemorrhage (VH) or PVR, the macular status (on or off), the location of main retinal tears (superior or inferior), the patient's age, and the refractive error. These clinical data were basically determined by the patient's medical records. The extent of detachment, macular status, and the location of main retinal breaks were estimated by the preoperative fundus sketch [7]. All of the enrolled participants were undergoing vitrectomy for the first time. Patients with RRD secondary to trauma or other disorders or those associated with diabetes mellitus were excluded. Since our earlier study examined the expression profiles of inflammatory cytokines in the vitreous fluid of PDR, RVO, ERM, or MH patients [4], we utilized these previously collected vitreous specimens for these disorders as the positive or negative controls in the present study.

### 2.2. Quantitative Analysis of Inflammatory Cytokines

Inflammatory cytokine concentrations were measured using a previously described method [4]. Briefly, vitreous fluid was diluted fourfold through the use of the dilution solution provided by the Bio-Plex® beads array kit (Bio-Rad Laboratories, Hercules, CA). Samples were prepared by first centrifuging the specimen at 10,000 ×g for 5 min after vortex agitation. A total of 50 *μ*L of supernatant was then used for the cytokine assay in accordance with the manufacturer's instructions. For the Bio-Plex kit, beads that had been separated by color for each target cytokine were conjugated with commercially obtained primary antibodies against the target cytokine. We measured the following 27 cytokines: IL-1*β*, IL-1 receptor antagonist (IL-1ra), IL-2, IL-4, IL-5, IL-6, IL-7, IL-8 [chemokine* C-X-C* motif ligand* (CXCL8)*], IL-9, IL-10, IL-12, IL-13, IL-15, IL-17, eotaxin [chemokine* C-C* motif ligand* (CCL11)*], basic fibroblast growth factor (bFGF), granulocyte-colony-stimulating factor (G-CSF), granulocyte/macrophage-colony-stimulating factor (GM-CSF), interferon- (IFN-) *α*, IFN-*γ*, MCP-1* (CCL2)*, MIP-1*α (CCL3)*, MIP-1*β (CCL4)*, IP-10* (CXCL10)*, platelet-derived growth factor (PDGF), regulated upon activation, normal T cell expressed and secreted (RANTES,* CCL5*), and VEGF. The measurements of these cytokines were performed by using 96-well assay plates and reagent kits according to the procedure provided by the manufacturer [4].

### 2.3. Statistics

Concentrations of cytokines were compared among the five groups (RRD, PDR, RVO, ERM, and MH). In order to characterize the expression profiles of the intravitreal cytokines, significant differences among the disease groups were at first analyzed by a one-way analysis of variance (ANOVA) conducted using the Kruskal-Wallis test. Subsequently, we then performed a post hoc Mann-Whitney *U* test to compare the differently expressed cytokines between the RRD and the PDR (positive control) and between the RD and the MH (nearly normal control). Normality of distribution in each group was examined by the Shapiro-Wilk test.

To analyze the interactions between the significantly upregulated cytokines in the vitreous of the RRD, Pearson's correlation coefficients were calculated between these cytokines. The significance level was examined by Student's* t*-test. To detect the most significantly correlated factors, we subsequently performed a stepwise multivariate regression analysis that took these significantly correlated cytokines into account.

To analyze the effect of the clinical features on the expressions of the cytokines, we first calculated Pearson's correlation coefficients between the cytokines and the clinical features. We then performed a stepwise multivariate regression analysis that specifically took into account the detached area (quadrants), duration of RRD (weeks), presence of VH or PVR, macular status, location of main retinal breaks, patient's age, and the refraction. All the statistical calculations were performed by SPSS version 22 (Statistical Package for the Social Sciences, Chicago, IL). *P* values < 0.05 were considered to be statistically significant.

## 3. Results

### 3.1. Expression Profiles of the Cytokines in RRD and Comparisons to Other Diseases

**Figure 1:**
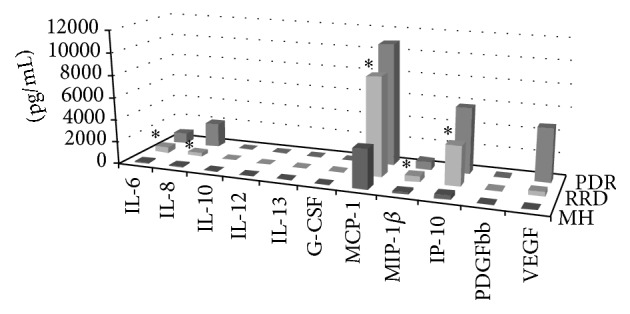
Kruskal-Wallis test showed there were different elevations in the expression profiles of the intravitreal cytokines for RRD, MH, PDR, ERM, and RVO. Although there were significantly greater upregulations of MCP-1, MIP-1*β*, and IP-10 in RRD versus MH (Mann-Whitney *U* test, *P* < 0.001, *P* = 0.041, and *P* < 0.001, resp.), there were no significant differences observed between RRD and PDR. IL-6 and IL-8 were significantly elevated to a greater degree in RRD versus MH (*P* = 0.009, *P* = 0.007, resp.), while they were significantly lower in RRD versus PDR (*P* = 0.037, *P* < 0.001, resp.). ERM and RVO data are not shown. *∗* indicates the cytokines which showed to be statistically significantly upregulated in the RRD vitreous compared with MH.

Table 1 lists the concentrations of the cytokines in the vitreous fluid that showed significant differences among disease groups (all data are presented in the Supplementary Table in the Supplementary Material available online at http://dx.doi.org/10.1155/2016/4256183). The data that are shown used median and quarterly variations, as some of data did not conform to the normal distribution. Cytokines that exhibited significant differences (Kruskal-Wallis test, *P* < 0.05) among the five diseases included IL-6 (*P* < 0.001), IL-8 (*P* < 0.001), IL-10 (*P* = 0.009), IL-12 (*P* = 0.006), IL-13 (*P* = 0.005), G-CSF (*P* = 0.026), MCP-1 (*P* < 0.001), MIP-1*β* (*P* = 0.012), IP-10 (*P* < 0.001), PDGF (*P* = 0.005), and VEGF (*P* < 0.001). The cytokines that were expressed in RRD at significantly higher levels versus MH included IL-6 (Mann-Whitney, *P* = 0.009), IL-8 (*P* = 0.007), MCP-1 (*P* < 0.001), MIP-1*β* (*P* = 0.041), and IP-10 (*P* < 0.001) (Figure 1). Comparisons of the cytokines between RRD and PDR showed that while the levels of IL-6 (*P* = 0.009) and IL-8 (*P* = 0.001) were significantly lower in RRD versus PDR, there were no statistical differences found for the levels of MCP-1 (*P* = 0.644), MIP-1*β* (*P* = 0.909), and IP-10 (*P* = 0.403). Even though VEGF is known to be elevated in PDR and RVO [4], there were no significant differences in the expression levels between RRD and MH (*P* = 0.058). However, the RRD did exhibit a significantly lower level of VEGF as compared to that observed for PDR (*P* < 0.001).

### 3.2. Interactions between Cytokines in RRD

**Figure 2:**
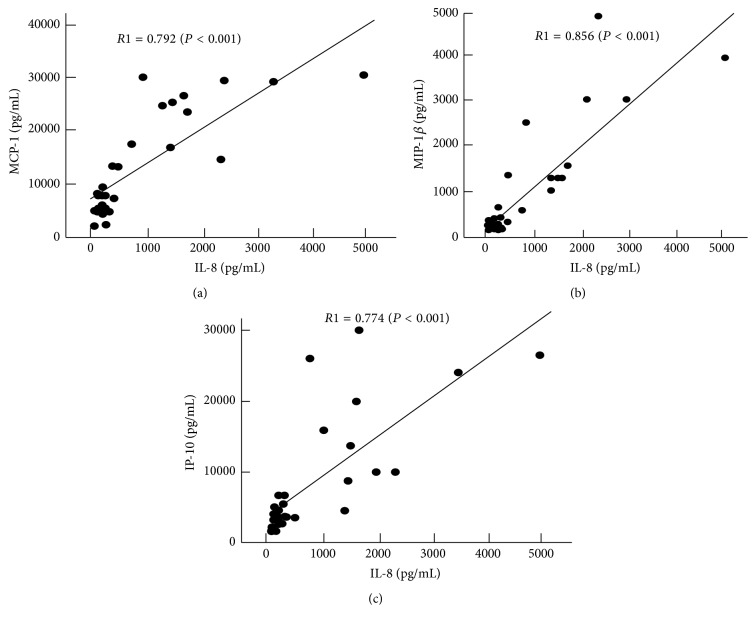
Significant correlations were observed between the IL-8 and the MCP-1 (a), MIP-1*β* (b), and IP-10 (c) in the RRD vitreous. *R*1 with a solid regression line indicates the correlation coefficient when the MH data are not included as a control.

Interactions between cytokines in the RRD vitreous were analyzed by calculating Pearson's correlation coefficient between each of the IL-6, IL-8, MCP-1, MIP-1*β*, and IP-10 pairs that exhibited a significantly higher expression in RRD versus MH. The results shown in Table 2 demonstrate that all of these cytokines were significantly correlated to each other. Moreover, MCP-1, MIP-1*β*, and IP-10 were all highly correlated with IL-8 Pearson's correlation coefficients (*R*1), with values of 0.792 (*P* < 0.001), 0.856 (*P* < 0.001), and 0.774 (*P* < 0.001), respectively (Figure 2). The stepwise multivariate regression analysis further indicated that MCP-1, MIP-1*β*, and IP-10 were the cytokines that were the most significantly correlated to IL-8 (standard partial regression coefficient, *b* = 0.792, 0.856, and 0.739, resp., *P* < 0.001 for each, Table 3).

### 3.3. Correlations between Cytokines and Clinical Features in RRD

**Figure 3:**
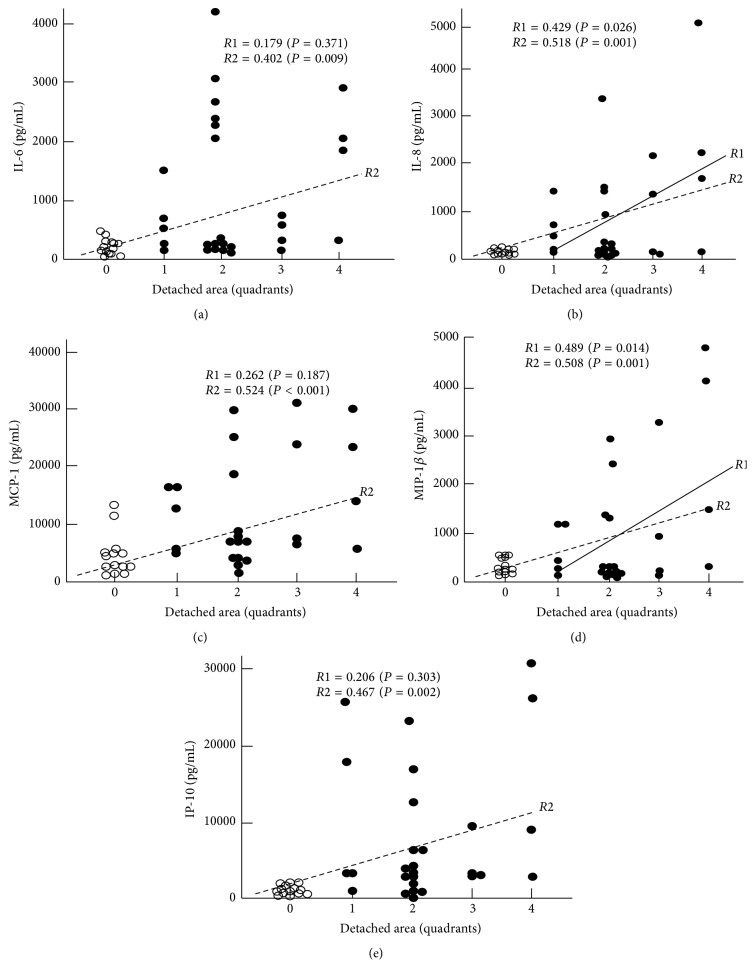
Correlations observed between the extent of RD and the IL-6, IL-8, MCP-1, MIP-1*β*, and IP-10. *R*1 with a solid regression line indicates Pearson's correlation coefficient when the MH data were not included, while *R*2 with a broken regression line indicates that the MH data were included as a detached area of the 0 quadrant.

**Figure 4:**
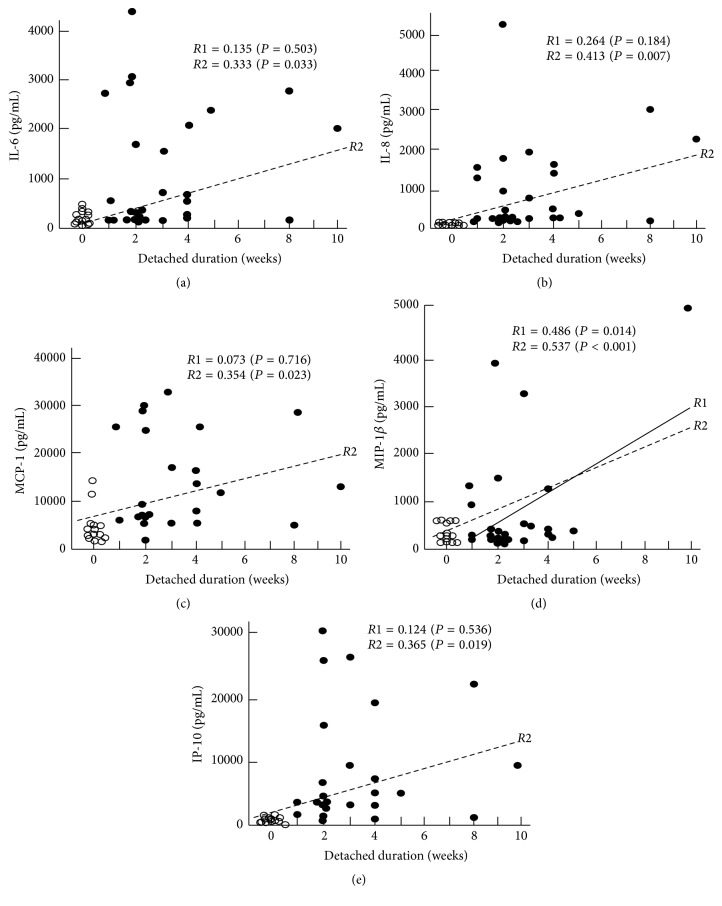
Correlations observed between the RRD and the IL-6, IL-8, MCP-1, MIP-1*β*, and IP-10 with the duration of RRD. *R*1 with a solid regression line means Pearson's correlation coefficient when data of MH are not included, while *R*2 with a broken regression line is that when the data of MH are included as a duration of the 0 week. While only *R*1 of MIP-1*β* exhibited a significant correlation to IL-8, statistical significance was observed for all of *R*2s.

Table 4 summarizes the correlation coefficients between the cytokines and the clinical features. The results show that IL-8 was significantly correlated to the detached area (*R*1 = 0.431, *P* = 0.022), while MIP-1*β* was significantly correlated to both the detached area (*R*1 = 0.489, *P* = 0.014) and the duration of the RRD (*R*1 = 0.486, *P* = 0.014). In addition, when the MH data were included as the detached area of the 0 quadrant and for the duration of the 0 week, the significance levels were increased and there was a significant correlation between these five cytokines and both the detached area (*R*2 in Figure 3) and the duration of RRD (*R*2 in Figure 4). The stepwise multivariate regression analysis (Table 5) also indicated that there was a significant correlation between IL-8 and the detached area (*b* = 0.443, *P* = 0.030) and that the MIP-1*β* was significantly correlated to both the detached area (*b* = 0.462, *P* = 0.012) and the duration of the RRD (*b* = 0.421, *P* = 0.020). The vitreous status, macular status, location of main retinal breaks, patient's age, and the refractive error showed no statistically significant correlations with the levels of these cytokines.

## 4. Discussion

The present study characterized the expression profiles of the inflammatory cytokines in the RRD vitreous. Our results revealed that MCP-1, MIP-1*β*, and IP-10 are significantly elevated in the RRD vitreous at levels that are indistinguishable from those seen in PDR. Moreover, the expressions of the IL-6 and IL-8 were significantly higher in the RRD versus the MH, while they were significantly lower in the RRD versus the PDR. In contrast, Abu El-Asrar et al. reported that the MCP-1, IL-8, and IP-10 were significantly upregulated in the vitreous in the PDR and PVR versus the RRD without PVR [5, 6]. This indicates a discrepancy with our current results, as 86% of our present RRD patients consisted of patients without PVR and VH. This may be, in part, due to the differences in the cytokine assay procedures. Even though VEGF is known to be upregulated in PDR and RVO [4], our study found that RRD did not exhibit any significantly higher level of VEGF other than for MH. Thus, our results appear to demonstrate a previously unknown specific pattern of intravitreal cytokines in RRD that differs from those which have been reported in MH, PDR, and other vitreoretinal diseases. Furthermore, we clarified that IL-6, IL-8, MCP-1, MIP-1*β*, and IP-10 were correlated to each other, with the most significant correlations observed between IL-8 and the MCP-1, MIP-1*β*, and IP-10. Our current results confirm our previous speculation that there is the presence of a network of inflammatory cytokines in the PDR and RVO vitreous [4].

In another of our previous studies, we also found that there might be an increase in the oxidative stress in the RRD vitreous [7]. Since IL-8 is known to be upregulated by oxygen stress [8], increased oxygen stress may induce the expression of IL-8 in RRD, thereby triggering the inflammatory reactions. Thus, we hypothesize that IL-8 may play an important role in the presumed cytokine network in RRD. In order to definitively clarify this hypothesis and the roles of proinflammatory cytokines like IL-8 and IL-6 in RRD, further investigations will need to be undertaken.

We additionally analyzed the correlations between the cytokine levels and the clinical features in an attempt to better understand the effects of the clinical factors on the inflammatory cytokine expression levels. Our analyses of the detached area, duration of RRD, presence of VH or PVR, macular status, location of main retinal breaks, age, and the refractive error indicated that the most significant correlations with the cytokine expression levels were for the detached area and the duration of the RRD. In particular, the IL-8 was significantly correlated to the detached area, while the MIP-1*β* was significantly correlated to both the detached area and the duration of the RRD. The other cytokines, such as IL-6, MCP-1, and IP-10, were only significantly correlated to these clinical features when MH was included as a control. Thus, this suggests that these three cytokines are also correlated to these clinical features, although the effects may be weaker than IL-8 and MIP-1*β*. Moreover, the amount of detached area and the duration of the RRD may also be related to the severity of the RRD that ultimately leads to the photoreceptor cell death and the development of fibrous proliferation through the upregulated cytokines. The photoreceptor cell death induces the release of intracellular molecules such as histones and high-mobility group box 1 proteins that are known to upregulate the expression of intravitreal IL-8 [9] and MCP-1 [10], respectively. Therefore, the increase of these intravitreal cytokine levels could indicate the level of presumed photoreceptor damage and could potentially have an important impact on the clinical field, as it is important to be able to preoperatively estimate the level of inflammation in the vitreous space in RRD patients.

Kunikata et al. [3] previously reported that MCP-1, MIP-1*β*, and IP-10 were significantly increased in the aqueous humor obtained from RRD patients and that these three cytokines were significantly correlated to each other. In the present study, we not only confirmed their results, but also confirmed that the most significant correlation in the vitreous was between the IL-8 and the MCP-1, MIP-1*β*, and IP-10, in addition to being correlated to each other. We previously determined that the VEGF was significantly correlated to IL-10, IL-13, IP-10, MCP-1, MIP-1*β*, and PDGF, but not to IL-6 and IL-8 in PDR [4]. Moreover, the subsequent multivariate regression analysis that we performed in this previous study revealed that the IL-10, IL-13, and PDGF were the factors that were the most significantly correlated to the VEGF level [4]. The results of the present study did not find similar tendencies in the RRD vitreous, even though we did find different correlations. Thus, the differences in these results suggest that there may be different mechanisms that produce the inflammatory reactions between RRD and PDR, with a greater influence exhibited by retinal ischemia. However, our present finding that VEGF was not significantly increased in the RRD vitreous indicates that the influence of ischemia in RRD can be considered to be remote.

Several research studies have suggested that inflammatory cytokines do have an effect on the photoreceptor damage in RRD. Nakazawa et al. examined an experimental RRD model and showed that MCP-1 was expressed in the Müller cells at 6 h after creation of the RRD and that a subretinal injection of MCP-1 induced photoreceptor apoptosis [1, 11]. MCP-1 has also been shown to promote the infiltration of monocytes in the detached retina, in addition to exhibiting photoreceptor toxicity via the infiltrated monocytes that are converted to macrophages or microglia [11]. MCP-1 has also been demonstrated to play a pivotal role in the development of chronic fibroproliferative diseases via persistent monocytes/macrophages [12–14]. All of these results imply that MCP-1 is one of the key factors in the development of PVR in RRD patients.

Although the role of MIP-1*β* in the detached retina has yet to be clarified and studied to the same degree as for MCP-1, MIP-1*β* has been reported to have an ability to induce migration of macrophages/microglia into an inflamed area [15] and to promote adhesion [16]. Therefore, both MCP-1 and MIP-1*β* in conjunction together may play a key role in causing the disease chronicity, thereby leading the development of a detached retina in PVR. Conversely, IP-10 is not only a chemoattractant to Th1-lymphocytes and monocytes, but it is also known to have angiostatic and antifibrotic effects [17, 18]. Therefore, we speculate that the upregulation of IP-10 in the RRD vitreous suggests the presence of compensatory mechanisms against the fibrogenesis promoted by MCP-1 and MIP-1*β*.

In conclusion, MCP-1, MIP-1*β*, and IP-10 are upregulated in the RRD vitreous at levels that are statistically indistinguishable from PDR, while IL-6 and IL-8 are only moderately elevated at levels that are significantly lower than that which is observed in PDR. However, all of these cytokines are positively correlated to each other, with the strongest correlation for IL-8 seen with MCP-1, MIP-1*β*, and IP-10. These results suggest the presence of a cytokine network in which IL-8 plays an important role. In addition, clinical features like the presence of a detached area and the duration of RRD may have an influence on the cytokine levels found in the RRD vitreous. In order to be able to better design proper treatments for those pathologic conditions, further analyses that lead to a better understanding of the role of inflammatory cytokines in the pathogenesis of the photoreceptor degeneration and the formation of PVR will need to be undertaken.

## Supplementary Material

Raw data of concentration of intravitreal cytokines in patients with RRD, MH, PDR, ERM, and RVO.

## Figures and Tables

**Table 1:** Concentrations of intravitreal cytokines showing statistical differences.

	RRD	MH	PDR	ERM	RVO	ANOVA,* P*	RRD-MH, *P*	RRD-PDR, *P*
IL-6	517.0 [1931.5]	77.3 [278.0]	918.0 [1703.0]	112.0 [303.75]	2116.5 [5464.0]	<0.001^*∗*^	0.009^*∗∗*^	0.037^*∗∗∗*^
IL-8	343.0 [1404.5]	94.5 [228.63]	2168.0 [3449.5]	168.0 [257.8]	4153.5 [12369.6]	<0.001^*∗*^	0.007^*∗∗*^	<0.001^*∗∗∗*^
IL-10	32.0 [47.0]	12.0 [41.4]	51.0 [60.0]	49.0 [40.0]	53.5 [86.25]	0.009^*∗*^	0.289	0.016^*∗∗∗*^
IL-12	16.0 [49.0]	12.5 [47.8]	51.0 [68.5]	53.5 [47.3]	68.5 [89.3]	0.006^*∗*^	0.460	0.005^*∗∗∗*^
IL-13	9.5 [43.5]	8.0 [34.6]	39.0 [47.0]	34.5 [44.3]	43.0 [62.1]	0.005^*∗*^	0.239	0.003^*∗∗∗*^
G-CSF	63.0 [77.0]	13.5 [50.9]	62.0 [68.5]	59.0 [55.3]	68.0 [173.4]	0.026^*∗*^	0.062	0.248
MCP-1	8740.0 [20034.0]	3471.3 [2930.6]	10892.0 [16580.5]	3132.0 [2318.5]	19843.0 [23562.8]	<0.001^*∗*^	<0.001^*∗∗*^	0.598
MIP-1*β*	478.0 [1202.0]	163.5 [495.8]	698.0 [921.5]	414.0 [387.0]	1170.8 [2237.6]	0.012^*∗*^	0.041^*∗∗*^	0.730
IP-10	3531.0 [11016.0]	390.0 [1091.8]	5858.0 [9544.5]	445.5 [2439.5]	10078.5 [22053.6]	<0.001^*∗*^	<0.001^*∗∗*^	0.367
PDGF	24.0 [49.0]	23.8 [43.4]	54.0 [90.5]	43.0 [65.5]	64.8 [113.9]	0.005^*∗*^	0.331	0.004^*∗∗∗*^
VEGF	355.0 [489.0]	67.0 [300.6]	4765.0 [11165.5]	260.5 [391.8]	3434.3 [20982.8]	<0.001^*∗*^	0.058	<0.001^*∗∗∗*^

Median [quartile deviation] is presented. The symbols *∗*, *∗∗*, and *∗∗∗* indicate statistically significant differences.

*∗*: ANOVA was conducted by Kruskal-Wallis test.

*∗∗*, *∗∗∗*: post hoc analyses were conducted by Mann-Whitney *U* test.

**Table 2:** Pearson's correlation coefficients between upregulated cytokines in the RRD vitreous.

	IL-8	IL-6	IP-10	MIP-1*β*
MCP-1	0.792 (*P* < 0.001)	0.686 (*P* < 0.001)	0.733 (*P* < 0.001)	0.709 (*P* < 0.001)
MIP-1*β*	0.856 (*P* < 0.001)	0.512 (*P* = 0.005)	0.583 (*P* = 0.001)	
IP-10	0.739 (*P* < 0.001)	0.653 (*P* < 0.001)		
IL-6	0.509 (*P* = 0.006)			

**Table 3:** Stepwise multivariate regression analysis between upregulated cytokines.

Explanatory variables	Dependent variables		
	MCP-1	MIP-1*β*	IP-10
	*b*	*b*	*b*
IL-8	0.792 (*P* < 0.001)	0.856 (*P* < 0.001)	0.739 (*P* < 0.001)

IL-6	0.303 (*P* = 0.026)	0.103 (*P* = 0.393)	0.373 (*P* = 0.012)
MCP-1	—	0.082 (*P* = 0.629)	0.396 (*P* = 0.066)
MIP-*β*	0.115 (*P* = 0.629)	—	−0.186 (*P* = 0.479)
IP-10	0.325 (*P* = 0.066)	−0.109 (*P* = 0.479)	—

*b*: standard partial regression coefficient.

**Table 4:** Pearson's correlation coefficients (*R*1) between cytokines and clinical features.

	Clinical features						
	Extent of RRD	Duration of RRD	VH or PVR	Macular status	Location of breaks	Age	Refractive error
IL-6	0.404 (*P* = 0.164)	0.164 (*P* = 0.405)	0.180 (*P *= 0.369)	−0.071 (*P* = 0.725)	0.152 (*P* = 0.478)	−0.031 (*P* = 0.877)	0.161 *(P* = 0.433)
IL-8	0.431 (*P* = 0.022)^*∗*^	0.245 (*P* = 0.209)	0.291 (*P* = 0.141)	0.111 (*P* = 0.583)	−0.250 (*P* = 0.239)	0.094 (*P* = 0.640)	0.139 (*P* = 0.497)
MCP-1	0.262 (*P* = 0.187)	0.073 (*P* = 0.716)	0.083 (*P* = 0.679)	−0.118 (*P *= 0.557)	−0.227 (*P* = 0.287)	0.147 (*P* = 0.465)	0.126 (*P* = 0.540)
MIP-1*β*	0.489 *(P* = 0.014)^*∗*^	0.486 (*P* = 0.014)^*∗*^	0.394 (*P* = 0.042)	0.006 (*P* = 0.977)	−0.264 (*P *= 0.213)	0.144 (*P* = 0.475)	0.137 (*P* = 0.506)
IP-10	0.208 (*P* = 0.287)	0.112 *(P* = 0.589)	0.293 (*P* = 0.138)	0.123 (*P* = 0.540)	−0.288 (*P* = 0.173)	−0.006 (*P* = 0.976)	0.023 (*P* = 0.910)

*R*1: correlation coefficient when MH is not considered as a control.

*∗*: statistically significant (*P* < 0.005).

**Table 5:** Stepwise multivariate regression analysis between cytokines and clinical features.

	IL-8	MIP-1*β*
	*b*	*b*
Extent of RRD	0.443 (*P* = 0.030)^*∗*^	0.462 (*P* = 0.012)^*∗*^
Duration of RRD	0.228 (*P* = 0.242)	0.421* (P* = 0.020)^*∗*^
PVR or VH	−0.379 (*P* = 0.076)	−0.344 (*P* = 0.069)
Macular status	−0.001 (*P* = 0.995)	−0.056 (*P* = 0.760)
Location of breaks	−0.116 (*P* = 0.580)	−0.134 (*P* = 0.760)
Age	0.062 (*P* = 0.757)	0.097 (*P* = 0.578)
Refractive error	0.310 (*P *= 0.122)	0.220 (*P* = 0.218)

*b*: standard partial regression coefficient.

*∗*: indicates statistically significant relationship.
