# Supplementary material for: Profiles of Inflammatory Cytokines in the Vitreous Fluid from Patients with Rhegmatogenous Retinal Detachment and Their Correlations with Clinical Features
Source: Biomed Res Int. 2016 Dec 15;2016:4256183. doi: 10.1155/2016/4256183 (PMC5198183; doi:10.1155/2016/4256183)
Supplement: Supplementary file 1 — Raw data of concentration of intravitreal cytokines in patients with RRD, MH, PDR, ERM, and RVO. [file 4256183.f1.pdf]

Supplementary Table. Concentrations of Intravitreal Cytokines

	RRD	MH	PDR	ERM	RVO	ANOVA, *P*	RRD-MH, *P*	RRD-PDR, *P*
IL-1β	34.5 [51.0]	13.5 [38.75]	41.0 [42]	30.5 [41.3]	60.3 [68.3]	0.265	0.515	0.338
IL-1ra	42.0 [72.0]	28.5 [53.3]	64.0 [63]	57.0 [62.3]	65.5 [123.8]	0.267	0.216	0.534
IL-2	39.0 [51.0]	34.8 [30.3]	43.0 [46.5]	37.0 [45.5]	51.5 [64.8]	0.811	0.828	0.594
IL-4	23.5 [87.0]	18.0 [79.1]	74.0 [73.0]	80.0 [75.3]	90.3 [100.1]	0.511	0.392	0.601
IL-5	28.0 [41.0]	4.8 [24.5]	23.5 [30.5]	21.0 [23.5]	25.8 [30.4]	0.136	0.041**	0.722
IL-6	517.0 [1931.5]	77.3 [278.0]	918.0 [1703.0]	112.0 [303.75]	2116.5 [5464.0]	< 0.001*	ʼ0.009**	0.037***
IL-7	26.0 [87.0]	20.3 [87.3]	66.0 [95.5]	74.0 [64.3]	80.0 [120.0]	0.239	0.919	0.065
IL-8	343.0 [1404.5]	94.5 [228.63]	2168.0 [3449.5]	168.0 [257.8]	4153.5 [12369.6]	< 0.001*	0.007**	< 0.001***
IL-9	114.0 [416.0]	117.5 [339.3]	258.0 [369.0]	365.0 [302.5]	311.0 [231.8]	0.505	0.331	0.450
IL-10	32.0 [47.0]	12.0 [41.4]	51.0 [60.0]	49.0 [40.0]	53.5 [86.25]	0.009*	0.289	0.016***
IL-12	16.0 [49.0]	12.5 [47.8]	51.0 [68.5]	53.5 [47.3]	68.5 [89.3]	0.006*	0.460	0.005***
IL-13	9.5 [43.5]	8.0 [34.6]	39.0 [47.0]	34.5 [44.3]	43.0 [62.1]	0.005*	0.239	0.003***
IL-15	1075.0 [543.0]	1183.3 [260.1]	1153.5 [390.5]	1095.0 [593.3]	1317.0 [952.3]	0.693	0.377	0.180
IL-17	56.5 [247.0]	53.5 [218.1]	213.5 [222.0]	240.5 [223.0]	198.8 [258.5]	0.902	0.874	0.760
eotaxin	22.0 [73.0]	15.3 [68.3]	72.0 [78.0]	55.0 [60.8]	62.5 [108.1]	0.052	0.633	0.018
bFGF	537.0 [545.3]	189.5 [541.0]	454.0 [525.0]	455.0 [560.8]	374.3 [475.1]	0.991	0.965	0.949
G-CSF	63.0 [77.0]	13.5 [50.9]	62.0 [68.5]	59.0 [55.3]	68.0 [173.4]	0.026*	0.062	0.248
GM-CSF	132.0 [641.0]	145.0 [629.3]	619.0 [666.5]	614.0 [585.3]	470.0 [649.8]	0.633	0.854	0.103
IFN-α	77.0 [233.0]	78.5 [197.1]	226.0 [234.0]	231.0 [207.3]	158.8 [252.6]	0.566	0.654	0.194
IFN-γ	20.0 [54.5]	12.5 [46.6]	43.5 [47.0]	51.0 [45.0]	81.8 [67.8]	0.271	0.228	0.468
MCP-1	8740.0 [20034.0]	3471.3 2930.6]	10892.0 [16580.5]	3132.0 [2318.5]	19843.0 [23562.8]	< 0.001*	< 0.001**	0.598
MIP-1α	56.5 [70.0]	14.0 [61.3]	54.5 [62.0]	66.0 [61.8]	89.3 [103.5]	0.185	0.331	0.540
MIP-1β	478.0 [1202.0]	163.5 [495.8]	698.0 [921.5]	414.0 [387.0]	1170.8 [2237.6]	0.012*	0.041**	0.730
IP-10	3531.0 [11016.0]	390.0 [1091.8]	5858.0 [9544.5]	445.5 [2439.5]	10078.5 [22053.6]	< 0.001*	< 0.001**	0.367
PDGF	24.0 [49.0]	23.8 [43.4]	54.0 [90.5]	43.0 [65.5]	64.8 [113.9]	0.005*	0.331	0.004***
RANTES	98.0 [240.5]	96.5 [101.4]	102.0 [301.5]	77.0 [270.8]	83.5 [287.5]	0.884	0.654	0.745
VEGF	355.0 [489.0]	67.0 [300.6]	4765.0 [11165.5]	260.5 [391.8]	3434.3 [20982.8]	< 0.001*	0.058	< 0.001***

Median [quartertile deviation] is presented.

* : statistically significant difference by ANOVA conducted by Kruskal-Wallis test.

, * : statistically significant differences by post hoc analysys conducted by Mann-Whitney U-test,
